# Enhanced radiobiological effects at the distal end of a clinical proton beam: *in vitro* study

**DOI:** 10.1093/jrr/rrt230

**Published:** 2014-05-13

**Authors:** Yoshitaka Matsumoto, Taeko Matsuura, Mami Wada, Yusuke Egashira, Teiji Nishio, Yoshiya Furusawa

**Affiliations:** 1 Research Center for Charged Particle Therapy, National Institute of Radiological Sciences, 4-9-1 Anagawa, Inage-ku, Chiba 263-8555, Japan; 2 Advanced Medical Sciences, Graduate School of Medicine, Hokkaido University, 15-7 Kita, Kita-ku, Sapporo, Hokkaido, 060-8638, Japan; 3 Research Center for Innovative Oncology, National Cancer Center Hospital East, 6-5-1 Kashiwanoha, Kashiwa, Chiba 277-8577, Japan

**Keywords:** proton beam, biological effectiveness, distal end, cell survival, spread-out Bragg-peak

## Abstract

In the clinic, the relative biological effectiveness (RBE) value of 1.1 has usually been used in relation to the whole depth of the spread-out Bragg-peak (SOBP) of proton beams. The aim of this study was to confirm the actual biological effect in the SOBP at the very distal end of clinical proton beams using an *in vitro* cell system. A human salivary gland tumor cell line, HSG, was irradiated with clinical proton beams (accelerated by 190 MeV/u) and examined at different depths in the distal part and the center of the SOBP. Surviving fractions were analyzed with the colony formation assay. Cell survival curves and the survival parameters were obtained by fitting with the linear–quadratic (LQ) model. The RBE at each depth of the proton SOBP compared with that for X-rays was calculated by the biological equivalent dose, and the biological dose distribution was calculated from the RBE and the absorbed dose at each position. Although the physical dose distribution was flat in the SOBP, the RBE values calculated by the equivalent dose were significantly higher (up to 1.56 times) at the distal end than at the center of the SOBP. Additionally, the range of the isoeffective dose was extended beyond the range of the SOBP (up to 4.1 mm). From a clinical point of view, this may cause unexpected side effects to normal tissues at the distal position of the beam. It is important that the beam design and treatment planning take into consideration the biological dose distribution.

## INTRODUCTION

Proton beam therapy is considered a new yet well-established modality of treatment for cancer and non-cancer diseases around the world [1–4]. The number of proton therapy facilities in the world, especially in Japan, has increased, and it has doubled within the last 10 years [5, 6]. More than 60 000 patients have been treated with proton beams, and high control rates for localized tumors have been reported [1–4, 7]. In recent years, advanced proton therapy [e.g. intensity-modulated proton therapy (IMPT)] has been adapted for irregularly shaped tumors, and the effect is beginning to examined by physical fundamental research [5, 6, 8, 9]. The International Commission on Radiation Units and Measurements (ICRU) recommends defining proton therapy doses as the product of the relative biological effectiveness (RBE) and the physical dose of the proton, with its unit as Gy [11, 12]. Recently, most clinical proton facilities have used a constant RBE value of 1.1, meaning that protons are assumed to be 10% more effective than X-rays or gamma-rays at all positions along the depth–dose distribution [11–14]. The RBE weighting factor of 1.1 was a consequence of several reviews of the available radiobiological data at those instances [12, 15, 16], with most studies determining the RBE in the center of SOBP. However, there is a general consensus that the RBE of protons depends on the position along the penetration depth [17–20]. Recent physical simulation results suggest the RBE is not constant and that it depends on many factors such as beam energy, dose, depth, radiation quality, and track structure [12, 21–23]. Additionally, modeling studies suggest that there are significant differences between the biologically weighted dose and the absorbed dose distributions for both tumor and normal tissues (using a theoretical variable RBE value to calculate an RBE-weighted proton treatment plan [24–26]). Although many studies have measured the RBE of protons, the experimental conditions were very diverse, with respect to differences in beam energy, position along the depth–dose distribution, method of calculating RBE, and cells used.

In this study, we have determined the RBE at various depths within the SOBP of clinical proton beams with an incident energy of 190 MeV, and have assessed the biological equivalent dose distribution of proton beams. We have also determined the shift of the distal edge of the biological dose compared with the isoeffective dose.

## MATERIALS AND METHODS

### Cell cultures

A human salivary gland tumor cell line, HSG (JCRB1070: HSGc-C5), was used in this study. The HSG is a standard reference cell line for the intercomparison of RBE among carbon and proton facilities in Japan, and is also used in other countries, including Germany and Korea [25, 27–32]. Cells were cultured in Eagle's MEM supplemented with 10% fetal bovine serum (FBS) and antibiotics (100 U/ml penicillin and 100 µg/ml streptomycin) and incubated under a humidified atmosphere with 5% CO_2_ and 95% air at 37°C. Subcultured cells were harvested and seeded in a chamber slide flask (Lab-Tech SlideFlask 170920, Nunc) at ∼1.5–2.0 × 10^5^ cells/flask with 3 ml of the medium, and incubated in the incubator for 2 d prior to the experiment. The flasks were fully filled with additional medium on the same day or 1 d before the experiment.

### Irradiation

**Fig. 1.**
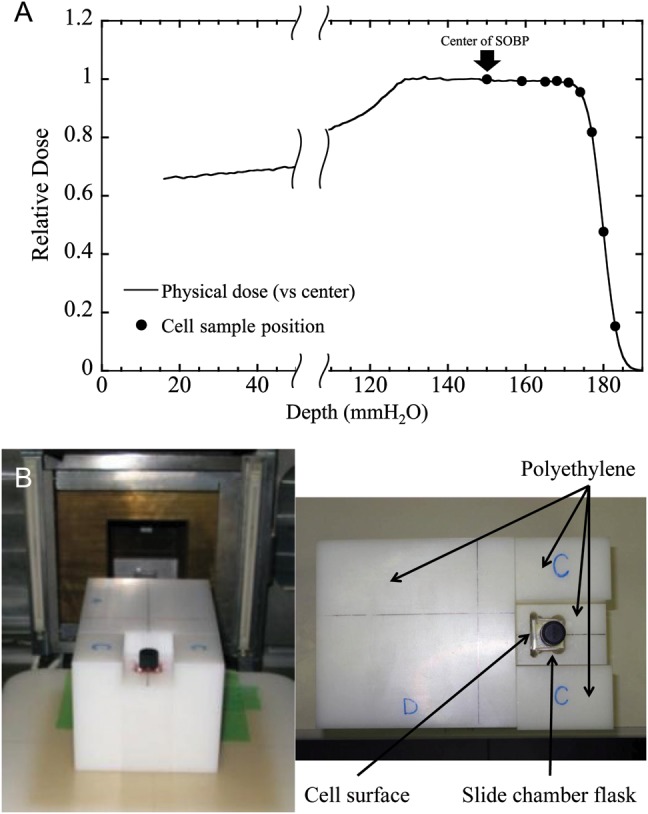
(**A**) Depth–dose distribution of the spread-out Bragg-peak (SOBP) of the 190 MeV proton beam used in the present experiment. The depth–dose measurement was performed in a water phantom. The closed dots show the irradiation position of each cell sample (150, 159, 165, 168, 171, 174, 177, 180 and 183 mmH_2_O). (**B**) The cell sample flask was placed in a specially designed polyethylene block (0.98 g/cm^3^) containing a space to hold it. The thickness of the polyethylene block in front of the flask was chosen to locate the cells at the adequate depth of the spread-out Bragg-peak (SOBP) beam.

Horizontal proton beams were accelerated up to 190 MeV by an Azimuthally Varying Field (AVF) cyclotron at the NCCHE (National Cancer Center Hospital East) [31]. In this experiment, we used the nozzle designed for the dual-ring scattering method [24] to obtain a flat dose profile and stable dose intensity over the target area. The proton beam was scattered using two thin scatters on the beam line. These scatters made it possible to obtain a flat dose profile over the target area (±2.5% over a 2 × 5 cm^2^ field). The beam was then cut off using collimators. The profile to the center position of the physical depth–dose distribution of the 5 cm-SOBP (from 125 to 175 mmH_2_O) was less than ±7.2% (Fig. 1A).

HSG cells on the bottom of the chamber slide flasks were set in a specially designed polyethylene block (0.98 g/cm^3^), and the cell surface was placed at the isocenter of the gantry (Fig. 1B). The depths (at 150, 159, 165, 168, 171, 174, 177, 180 and 183 mmH_2_O) in the beam were selected using polyethylene blocks of various thicknesses placed immediately upstream of the cells. The measurement of the dose and dose-rate was conducted with PTW Markus Chamber (Type 23343; PTW, Freiburg, Germany) and an electrometer (FLUKE35040; Fluke Biomedical, Cleveland, OH). Subsequently, GafChromic EBT film (International Specialty Products, Wayne, NJ) was used for verification. We also measured the dose per monitor unit at the center of the SOBP, and used the average value calculated from at least three measurements on each experimental day. The dose rate was ∼2–3 Gy/min at each depth.

As for the reference radiation beam, 6 MV X-rays generated by a linac therapy machine at the NIRS (National Institute of Radiological Sciences) were used. The irradiation doses were measured with the thimble chamber according to the protocol of Japanese Standard Dosimetry 01 [33] for X-rays. The dose rate was ∼3 Gy/min. All irradiation was carried out at room temperature, and all experiments were repeated at least three times. X-ray experiments were performed as additional experiments at NIRS, because the treatment schedule of NCCHE was crowded. However, X-ray experiments were performed under the same conditions (i.e. lot and passage number of cells, sample preparation, and assay environment) as the proton beam experiment. We suspect the error caused by carrying out the proton and X-ray beam experiments on different day is not significant.

### *In vitro* clonogenic cell survival assay

After irradiation, cells were rinsed twice with PBS, once with 0.05% trypsin solution containing 1 mM EDTA and maintained at 37°C for 3–5 min. The cells were harvested and their number counted using a particle analyzer (Coulter Z1). The cells were then adequately diluted with the medium and seeded in three 60-mm dishes at densities from 100–50 000 cells per dish to yield ∼100 colonies per dish, depending on the radiation dose and the linear energy transfer (LET). Three colony dishes were made per dose within one experiment. Samples were incubated for 13 d, and then the colonies were rinsed with PBS, fixed with 10% formalin solution for 10 min, washed with tap water, stained with 1% methylene blue solution, and dried in air. Colonies consisting of > 50 cells were counted under a stereomicroscope as the number of viable cells.

### Analysis of the survival curve

**Fig. 2.**
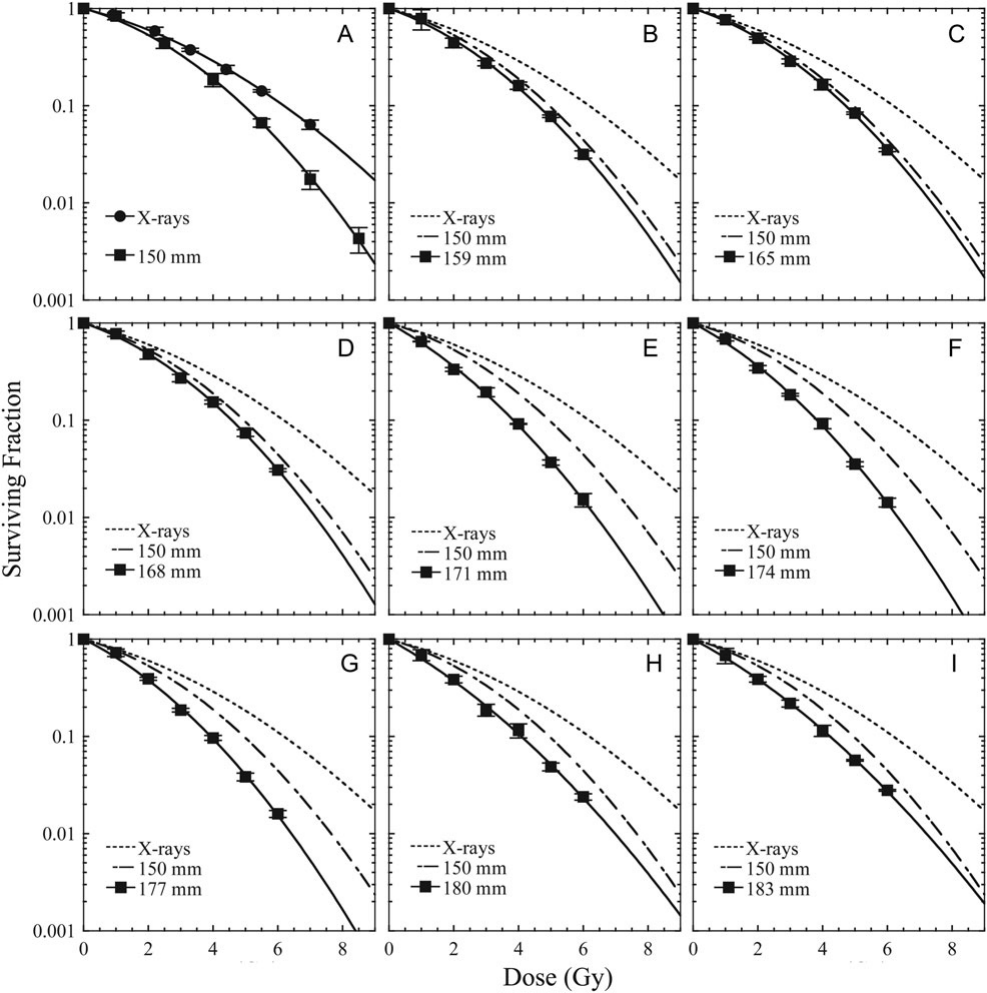
Dose–response curves of HSG cells irradiated with X-rays (closed circles) or at each depth of the proton SOBP (closed squares). All datapoints were fitted by the linear–quadratic (LQ) model. The symbols and bars are the mean and standard error (SE) obtained from at least three independent experiments. The symbols and bars of the reference X-rays data and the center of the SOBP as a reference data are indicated only in Fig. 3A, and the fitted curves (dotted line for X-rays and dashed line for center of the SOBP) for the various positions are indicated in Fig. 3B–I. Horizontal axis, Dose (Gy) means the physical absorbed dose of X-rays and protons at each depth.

Dose–response curves of HSG cells were fitted by a linear–quadratic equation. The parameters *α* and *β* were calculated by logistic curve-fitting using the weighted least-squares method (Kaleida Graph 4.1.4, Hulinks). The *α* and *β* values were used to calculate the biological equivalent doses, *D*_10_ and *D*_60_ values, the dose required for the cell survival to be 10 and 60%, respectively. *D*_60_ corresponds approximately to the survival fraction for 2 Gy X-rays for this cell type (Fig. 2 and Table 1). The RBE_10_ and RBE_60_ values of the proton beam were calculated as the ratio of the *D*_10_ and *D*_60_ values to that of 6 MV X-rays.

**Table 1.** The surviving parameters and biological equivalent dose for HSG cells in a 190 MeV clinical proton beam with a 5-cm spread-out Bragg-peak (SOBP) at each depth compared with the center

	X-rays	Proton								
		Depth in H_2_O (mm)								
		150	159	165	168	171	174	177	180	183
*α* (Gy^−1^)	0.19 ± 0.03^a^	0.22 ± 0.07	0.28 ± 0.07	0.25 ± 0.04	0.26 ± 0.03	0.42 ± 0.01	0.41 ± 0.04	0.38 ± 0.03	0.44 ± 0.06	0.42 ± 0.07
*β* (Gy^−2^)	0.03 ± 0.01	0.05 ± 0.01	0.05 ± 0.01	0.05 ± 0.01	0.05 ± 0.01	0.05 ± 0.01	0.05 ± 0.01	0.05 ± 0.01	0.03 ± 0.01	0.03 ± 0.01
*α*/*β* (Gy)	6.82 ± 2.83	4.40 ± 0.85	5.85 ± 2.60	5.08 ± 1.25	5.00 ± 1.01	9.12 ± 0.91	8.44 ± 2.04	7.34 ± 1.49	14.9 ± 5.56	15.6 ± 7.15
SF_2_	0.60 ± 0.01	0.53 ± 0.01	0.48 ± 0.03	0.49 ± 0.02	0.48 ± 0.01	0.36 ± 0.01	0.36 ± 0.01	0.38 ± 0.01	0.37 ± 0.02	0.39 ± 0.02
*D*_10_ (Gy)	6.12 ± 0.15	4.93 ± 0.16	4.60 ± 0.11*	4.70 ± 0.07*	4.57 ± 0.08**	3.84 ± 0.07**	3.82 ± 0.04**	3.92 ± 0.08**	4.06 ± 0.14**	4.21 ± 0.11**
*D*_60_ (Gy)	2.02 ± 0.12	1.68 ± 0.11	1.48 ± 0.18	1.55 ± 0.11*	1.50 ± 0.10**	1.08 ± 0.02**	1.10 ± 0.06**	1.16 ± 0.06**	1.09 ± 0.11**	1.14 ± 0.14**

The surviving parameters (*α*, *β* and *α*/*β*) were obtained from fitting the survival data with a linear–quadratic equation, and SF_2_, *D*_10_ and *D*_60_ values were calculated using these parameters. ^a^ Mean ± standard error. *0.01 < *P* < 0.05, **P < 0.01, compared with the center of proton SOBP samples.

### Statistical analysis

Data are presented as the mean ± standard error (SE) of at least three independent experiments. To examine the differences between averages of values, a two-sided Student's *t*-test was used when the variances of two groups could be assumed to be equal. A *P*-value < 0.05 was considered statistically significant.

## RESULTS

### Survival of HSG cells exposed to proton beams at several depths

The dose–response curves for HSG cells to X-rays and proton beam SOBPs at each depth are shown in Fig. 2. The *D*_10_ and *D*_60_ values were calculated from the *α* and *β* values (Table 1). The SOBP beam at the center (150 mmH_2_O) killed HSG cells more efficiently than the X-rays (Fig. 2A), and the effects increased gently from 159 to 168 mmH_2_O in the SOBP beam (Fig. 2B–D, Table 1) compared with at the center. However, the cytotoxic effects increased dramatically after 171 mmH_2_O, and the survival curves were similar to each other at higher values (Fig. 2E–I, Table 1).

For HSG cells, the *α* values of protons tended to be larger than for that of linac X-rays, while the *β* values tended to remain stable. The *α*/*β* ratio was 6.8 Gy for X-rays. The value once decreased in the 150–168 mmH_2_O region (4–6 Gy), increased a little (7–9 Gy) in the 171–177 mmH_2_O region, and increased suddenly (approximately 15 Gy) at 180 and 183 mmH_2_O (Table 1).

### Change of RBE in SOBP

**Fig. 3.**
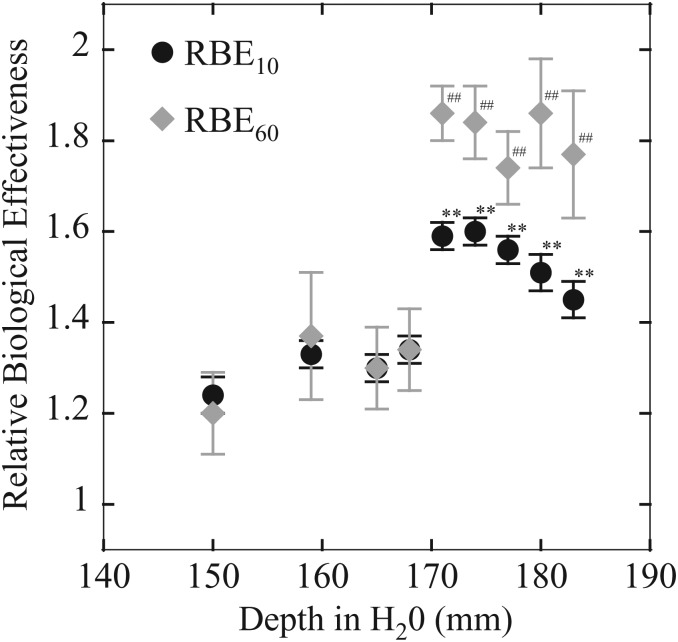
Relative biological effectiveness (RBE) for HSG cells in a 190 MeV clinical proton beam with a 5-cm spread-out Bragg-peak (SOBP). RBE_10_ (closed circles) and RBE_60_ (closed diamonds) represent the RBE calculated using the biological equivalent dose, *D*_10_ and *D*_60_, respectively. The symbols and bars are the mean and standard error (SE) obtained from at least three independent experiments. *0.01 < *P* < 0.05, **P < 0.01, compared with the center of the proton SOBP samples using RBE_10_. ^#^0.01 < *P* < 0.05, ^##^*P* < 0.01, compared with the center of the proton SOBP samples using RBE_60_.

The RBE_10_ and RBE_60_ to the depth in SOBP that correspond to *D*_10_ and *D*_60_ are shown in Fig. 3. *D*_10_ values are commonly used to compare the cytotoxic effects of radiation types. HSG cells presented RBE_10_ values of 1.24 and RBE_60_ values of 1.20 at the center of proton SOBP. The RBE values showed a tendency to increase with the depth of proton SOBP, and the maximum value was 1.86 at 180 mmH_2_O. These values mean that the proton SOBP beam showed ∼50% stronger cytotoxic effects at the distal position compared with at the center of the SOBP.

**Fig. 4.**
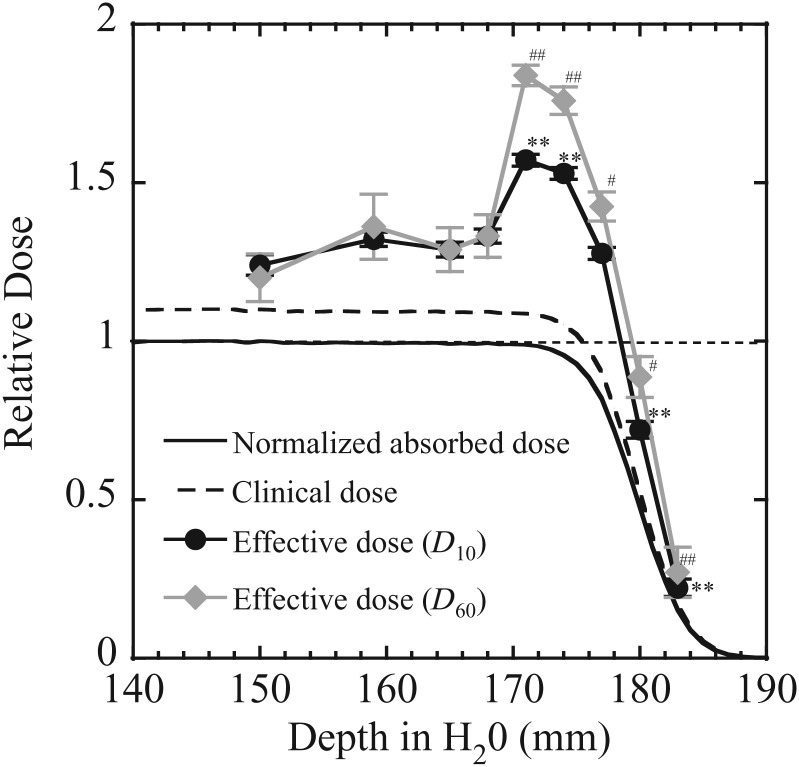
Relative dose–depth distribution of the spread-out Bragg-peak (SOBP) of the 190 MeV proton beam. The biological dose was calculated as the relative biological effectiveness (RBE) × relative physical dose at each depth compared with the center. Effective dose (*D*_10_) (closed circles) and effective dose (*D*_60_) (closed diamonds) represents the biological dose calculated by the RBE_10_ and RBE_60_, respectively. The symbols and bars are the mean and standard error (SE) obtained from at least three independent experiments. The solid line indicates the relative physical absorbed dose normalized with the center dose, and the dotted line indicates the clinical dose calculated from the generic proton RBE 1.1 × physical dose at each depth. *0.01 < *P* < 0.05, **P < 0.01, compared with the center of the proton SOBP samples using the effective dose (*D*_10_). ^#^0.01 < *P* < 0.05, ^##^*P* < 0.01, compared with the center of the proton SOBP samples using the effective dose (*D*_60_).

The depth–dose distributions are shown in Fig. 4. The normalized absorbed dose refers to the relative physical dose normalized to the center of the SOBP. A generic RBE value of 1.1 for protons is used in clinical situations, and an Isoeffective dose *D*_IsoE_ = *D* × 1.1 is proposed [11]. The profile of the biological effective dose in this paper can be calculated from the RBE_10_ or RBE_60_ at each depth multiplied by the physical dose at that depth. The biological effective doses at the center of the SOBP were slightly higher than the isoeffective dose (Fig. 4). The values of biological effective doses were not significantly changed between 150 and 168 mmH_2_O, significantly increased at a depth of 171 to 177 mmH_2_O, then decreased with decrease of physical dose, however the biological effective dose was still higher than the isoeffective dose at 180 and 183 mmH_2_O. Additionally, in the current study, the distal edge of the biological dose was extended ∼3.6 mm for RBE_10_ and 4.1 mm for RBE_60_ from the edge of SOBP obtained by the isoeffective dose.

## DISCUSSION

Here, we have reported the RBE dependence of the biological depth–dose distribution at several depth positions of 190 MeV proton beams accelerated by a cyclotron and in the SOBP generated by the dual-ring scattering method. A generic RBE of 1.1 is recommended for the whole region of proton SOBPs for all clinically relevant applications worldwide [11, 13, 14, 34]. Therefore, all clinical applications are conducted at that RBE value, and the flat adsorbed depth–dose distribution is used in the therapies. However, some studies have demonstrated an increase in the RBE at the end of the proton SOBP using physical simulations [22, 23] and analysis of published biological results [11]. Wilkens *et al*. reported that the RBE of the distal region of a SOBP increased to 1.18–1.60 depending on the fraction size of 1–8 Gy per fraction in the case of 160-MeV protons [22]. In the present study, the RBE values varied with depth and were higher at the distal-end of the SOBP for 190 MeV clinical protons. This result suggests that it is necessary to set the absorbed depth–dose distribution according to the differences in the biological effect. The RBE of protons could depend on the fraction size. The conventional fractionation scheme of proton therapy is ∼2 GyE (generic RBE 1.1 × physical dose) per fraction [35]. The effective doses of the proton SOBP that correspond to 2 Gy X-rays were calculated using the *α* and *β* values. RBE values of proton SOBP beams at 60% cell survival were defined as RBE_60_, because the surviving fraction at 2 Gy SF_2_ of X-rays is ∼0.6. The RBE_60_ for HSG cells showed a maximum value of 1.56 at the distal end of the SOBP (Table 1). High-LET components could be effective on cells with small *α* and *α*/*β* values [28]. These high-LET components account for a large part of the total proton beams at the distal position in the SOBP, even after the decay of the SOBP when most of the beams lose energy. This could be the reason for the higher RBE values at 171–183 mmH_2_O than at 150–168 mmH_2_O positions.

Additionally, the more critical point in clinical settings is the shift of the distal edge of the biological dose compared with the isoeffective dose. According to the strong biological effect at the distal region of the proton SOBP, the biological depth–dose distribution may be extended to the direction of the proton beam prediction calculated by the generic RBE 1.1. In fact, unexpected normal tissue damage caused by the beam is rarely observed in the clinical field of proton therapy (personal communication with Dr Kanemoto, Proton Medical Research Center, Tsukuba University). According to a phenomenological model, one previous report showed that the distal edge of the biological dose was shifted from 1.1 to 2.2 mm for 80% physical dose points at 1–8 Gy [22]. Our results yielded 4.1 mm from the isoeffective dose at 2 Gy (Fig. 4). There are similarities between Wilkens's study and our results. The distal shift could have been altered by the local energy distribution of the protons at the cells/tissues caused by the accelerated energy or the geometrical structure of the instruments upstream of the target. This study has certain limitations. We assessed the biological effects using one cell line (HSG cells) and one biological endpoint (cell survival). However, it is well known that the RBE values change depending on the kind of cells and endpoints [36]. Therefore, in any further study we will have to assess the biological effects using another cell line, other experimental animals and another biological technique (e.g. DNA repair, chromosomal aberration, mutation) [37–39].

There is also a problem regarding the use of the HSG cells. We chose HSG cells in this study because we have used HSG cells from the same cell line in experiments in particle beam facilities since the 1990s. Additionally, many laboratories (including ours) have used these cells in research and published many papers in international journals. However, it has been reported that the HSG cells used in this study were contaminated with HeLa cells [40, 41]. It will be necessary to consider the alternative cell line used for the particle beam facility experiments in the future.

## CONCLUSION

In conclusion, the effective depth–dose distribution was not flat in the proton SOBP. RBE_10_ and RBE_60_ at the distal region of the SOBP showed a maximum of 1.5 and 1.7 at the 10% and 60% survival level, respectively. The uniform biological dose region at 90% of the prescribed dose extended to 3.6 and 4.1 mm, respectively. Distal-end regions of proton beams are characterized with high effectiveness, and the SOBP range may be extended by several mm in the direction of the beam. We suggest that it is desirable to take into consideration the biological dose distribution according to the depth in beam design and treatment planning, however, further research is crucial.

## FUNDING

This work was supported in part by the Special Coordination Funds of President for Research Projects with Heavy Ions at the National Institute of Radiological Sciences–Heavy-ion Medical Accelerator in Chiba (NIRS–HIMAC).
